# Addition of K22 Converts Spider Venom Peptide Pme2a from an Activator to an Inhibitor of Na_V_1.7

**DOI:** 10.3390/biomedicines8020037

**Published:** 2020-02-19

**Authors:** Kathleen Yin, Jennifer R. Deuis, Zoltan Dekan, Ai-Hua Jin, Paul F. Alewood, Glenn F. King, Volker Herzig, Irina Vetter

**Affiliations:** 1 Centre for Health Informatics, Australian Institute of Health Innovation, Macquarie University, North Ryde, NSW 2109, Australia; kathleen.yin@mq.edu.au; 2 Institute for Molecular Bioscience, The University of Queensland, St. Lucia, QLD 4072, Australia; j.deuis@uq.edu.au (J.R.D.); z.dekan@imb.uq.edu.au (Z.D.); p.alewood@imb.uq.edu.au (P.F.A.); glenn.king@imb.uq.edu.au (G.F.K.); 3 School of Science & Engineering, University of the Sunshine Coast, Sippy Downs, QLD 4556, Australia; 4 School of Pharmacy, The University of Queensland, Woolloongabba, QLD 4102, Australia

**Keywords:** sodium channel, Na_V_1.7, Na_V_1.8, venom, spider, peptide

## Abstract

Spider venom is a novel source of disulfide-rich peptides with potent and selective activity at voltage-gated sodium channels (Na_V_). Here, we describe the discovery of μ-theraphotoxin-Pme1a and μ/*δ*-theraphotoxin-Pme2a, two novel peptides from the venom of the Gooty Ornamental tarantula *Poecilotheria metallica* that modulate Na_V_ channels. Pme1a is a 35 residue peptide that inhibits Na_V_1.7 peak current (IC_50_ 334 ± 114 nM) and shifts the voltage dependence of activation to more depolarised membrane potentials (V_1/2_ activation: Δ = +11.6 mV). Pme2a is a 33 residue peptide that delays fast inactivation and inhibits Na_V_1.7 peak current (EC_50_ > 10 μM). Synthesis of a [+22K]Pme2a analogue increased potency at Na_V_1.7 (IC_50_ 5.6 ± 1.1 μM) and removed the effect of the native peptide on fast inactivation, indicating that a lysine at position 22 (Pme2a numbering) is important for inhibitory activity. Results from this study may be used to guide the rational design of spider venom-derived peptides with improved potency and selectivity at Na_V_ channels in the future.

## 1. Introduction

Voltage-gated sodium channels (Na_V_) are pore-forming transmembrane proteins that regulate the influx of Na^+^ ions across excitable cell membranes, making them essential for the initiation and propagation of action potentials. In humans, nine different subtypes have been described (Na_V_1.1-1.9), each with unique biophysical properties and tissue-specific expression profiles [1]. Several subtypes, including Na_V_1.7 and Na_V_1.8, are highly expressed in peripheral sensory neurons and are therefore critical for somatosensation and nociception.

Na_V_1.7 and Na_V_1.8 are almost exclusively expressed in the peripheral nervous system, with preferential expression in small-diameter unmyelinated nociceptive or “pain-sensing” neurons [2,3,4]. In humans, loss-of-function mutations in *SCN9A*, the gene encoding Na_V_1.7, leads to congenital insensitivity to pain, while several gain-of-function mutations in *SCN9A* and *SCN10A* (the gene encoding Na_V_1.8) are associated with painful peripheral neuropathies [5,6,7]. This is consistent with studies in rodents, whereby knockout of *Scn9a* or *Scn10a* leads to deficits in mechanical, thermal and inflammatory pain [8,9], making both Na_V_1.7 and Na_V_1.8 promising therapeutic targets of interest for the treatment of pain. 

While many small-molecule Na_V_ inhibitors are used in the clinic, non-selective activity over other Na_V_ subtypes, including the central isoforms Na_V_1.1 and Na_V_1.2 and the cardiac specific isoform Na_V_1.5 [10,11], limits their widespread use as analgesics. Therefore, there has been a push to develop novel Na_V_ inhibitors with improved subtype selectivity that target the less conserved voltage-sensing domains of the channel [12]. One source of novel Na_V_ modulators is venoms from spiders, cone snails and scorpions, from which many peptides with exquisite Na_V_ subtype selectivity have been described [13,14]. However, compared to tetrodotoxin (TTX)-sensitive Na_V_ subtypes, relatively few venom-derived peptides with sub-micromolar potency at Na_V_1.8 have been characterised [15]. 

Therefore, the aim of this study was to use a high-throughput screen at Na_V_1.8 to identify novel bioactives from spider venom. Here, we describe the isolation and characterisation of two novel peptides from the Gooty Ornamental tarantula *Poecilotheria metallica* that modulate sodium channels. 

## 2. Experimental Section

### 2.1. Cell Culture

Human Embryonic Kidney (HEK) 293 cells stably expressing human Na_V_1.7/β1 (SB Drug Discovery, Glasgow, UK) were cultured in Minimum Essential Medium (MEM) supplemented with 10% fetal bovine serum (FBS), 2 mM l-glutamine, and the selection antibiotics G-418 (0.6 mg/mL) and blasticidin (4 μg/mL), as recommended by the manufacturer. Chinese Hamster Ovary (CHO) cells stably expressing human Na_V_1.8/β3 in a tetracycline-inducible system (ChanTest, Cleveland, OH, USA) were cultured in MEM supplemented with 10% FBS and 2 mM l-glutamine. Expression of hNa_V_1.8 was induced by the addition of tetracycline (1 μg/mL) for 48–72 h prior to assays. HEK293 cells stably expressing rat transient receptor potential vanilloid 1 (TRPV1) were cultured in Dulbecco’s Modified Eagle Medium (DMEM) containing 10% FBS under selection with hygromycin B (100 μg/mL), generated as previously described [16]. Cells were grown in an incubator at 37 °C with 5% CO_2_ and passaged every 3–4 days (at 70%–80% confluency) using TrypLE Express (Thermo Fisher Scientific, Scoresby, VIC, Australia). 

### 2.2. Venom Collection

Venom of female *P. metallica* spiders was extracted by weak electrical stimulation as previously described [17] and dried and stored at −20 °C, before being pooled and redissolved in milliQ water for further analysis.

### 2.3. Membrane Potential Assay at Na_V_1.8

CHO cells stably expressing hNa_V_1.8/β3 were plated 48 h before the assay on 384-well black-walled imaging plates coated with CellBIND (Corning, MA, USA) at a density of 10,000–15,000 cells/well and loaded with red membrane potential dye (Molecular Devices, Sunnyvale, CA, USA) plus TTX (1 μM) diluted in physiological salt solution (PSS; 140 mM NaCl, 11.5 mM glucose, 5.9 mM KCl, 1.4 mM MgCl_2_, 1.2 mM NaH_2_PO_4_, 5 mM NaHCO_3_, 1.8 mM CaCl_2_, 10 mM HEPES) and incubated for 30 min at 37 °C. Crude dried venom (10 μg/well) was diluted in PSS with 0.1% bovine serum albumin (BSA) and added using the FLIPR^TETRA^ (Molecular Devices) and incubated for 5 min before activation of Na_V_1.8 by addition of deltamethrin (150 μM). Changes in membrane potential were assessed using the FLIPR^TETRA^ (excitation, 515–545 nm; emission, 565–625 nm) every 2 s for 25 min after adding the agonist. To quantify the activity of crude venom at Na_V_1.8, the area under the curve (AUC) after the addition of deltamethrin was computed using ScreenWorks (Molecular Devices, Version 3.2.0.14). 

### 2.4. Isolation of Pme1a and Pme2a 

Crude *P. metallica* venom (1 mg dried mass) was dissolved in 5% acetonitrile (ACN)/0.1% trifluoroacetic acid (TFA) and loaded onto an analytical C_18_ Reversed-Phase (RP) High-Pressure Liquid Chromatography (HPLC) column (Vydac 4.6 × 250 mm, 5 μm; Grace, Columbia, MD, USA) attached to an UltiMate 3000 HPLC system (Dionex, Sunnyvale, CA, USA). Venom fractions were collected in 1 min intervals eluting at a flow rate of 0.7 mL/min with solvent A (0.1% formic acid in H_2_O) and solvent B (90% ACN, 0.1% formic acid in H_2_O) using the gradient: 5% solvent B over 5 min, followed by 5%–50% solvent B over 45 min followed by 50%–100% solvent B over 25 min.

Venom fractions were assessed for activity at hNa_V_1.8 using the FLIPR^TETRA^ membrane potential assay as described above. The active fraction was further fractionated to near-purity and peptide masses were determined using matrix-assisted laser desorption/ionization time-of-flight (MALDI-TOF) mass spectrometry (MS) using a Model 4700 Proteomics Analyser (Applied Biosystems, Foster City, CA, USA) with α-cyano-4-hydroxycinnamic acid (7 mg/mL in 50% ACN + 5% formic acid in H_2_O) as the matrix. Peptide sequences were determined by Edman degradation performed by the Australian Proteome Analysis Facility (Macquarie University, NSW, Australia). Sequence ambiguity was clarified by MS/MS sequencing. 

### 2.5. Peptide Synthesis

Peptides (Pme1a, Pme2a and [+K22]Pme2a) were assembled using a Symphony (Protein Technologies Inc., Tuscon, AZ, USA) automated peptide synthesizer on Fmoc-Rink-amide polystyrene resin on 0.1 mmol scale. Amino acid sidechains were protected as Asn(Trt), Arg(Pbf), Asp(OtBu), Cys(Trt), Gln(Trt), Glu(OtBu), His(Trt), Lys(Boc), Ser(tBu), Thr(tBu), Trp(Boc) and Tyr(tBu). Fluorenylmethyloxycarbonyl (Fmoc) removal was achieved using successive treatments with 30% piperidine/dimethylformamide (DMF) of 1 min then 3 min. Couplings were performed using 5 equivalents of HCTU/Fmoc-amino acid/N,N-diisopropylethylamine (DIEA) (1:1:1) in DMF, repeated twice (4 min then 8 min). Peptide-resins were cleaved using 3% triisopropylsilane (TIPS)/3% H_2_O/TFA for 2 h. Following evaporation of TFA under a stream of nitrogen, peptides were precipitated and washed with cold diethyl ether, dissolved in 50% ACN/0.1 % TFA/H_2_O and lyophilised. Crude peptides were purified by preparative RP-HPLC. Oxidative folding was performed in the presence of oxidised and reduced glutathione for 2 day at 4 °C, and the major folded products of correct mass were isolated by preparative RP-HPLC. 

### 2.6. Calcium Responses in TRPV1-HEK Cells

HEK cells stably expressing rTRPV1 were plated 48 h before the assay on 384-well black-walled imaging plates coated with poly d-lysine (ViewPlate-384, Perkin Elmer, Victoria, Australia) at a density of 10,000–15,000 cells/well and loaded with Calcium 4 no-wash dye (Molecular Devices) diluted in PSS and incubated for 30 min at 37 °C. Capsaicin and Pme1a were diluted in PSS with 0.1% bovine serum albumin (BSA) at the concentrations stated and added using the FLIPR^TETRA^. Changes in fluorescence were assessed using the FLIPR^TETRA^ (excitation 470–495 nm, emission 515–575 nm) every 1 s for 300 s after the addition of compounds. Fluorescence responses were quantified by the maximum increase in fluorescence calculated using ScreenWorks 3.2.0.14. 

### 2.7. Electrophysiology

Whole-cell patch-clamp experiments in Na_V_1.7-HEK cells and Na_V_1.8-CHO cells were performed on a QPatch-16 automated electrophysiology platform (Sophion Bioscience, Ballerup, Denmark) as previously described [18]. The extracellular solution contained in mM: NaCl 140, KCl 4, CaCl_2_ 2, MgCl_2_ 1, HEPES 10 and glucose 10; pH 7.4; osmolarity 305 mOsm. The intracellular solution contained in mM: CsF 140, EGTA/CsOH 1/5, HEPES 10 and NaCl 10; pH 7.3 with CsOH; osmolarity 320 mOsm. Concentration–response curves were acquired using a holding potential of −90 mV and a 50 ms pulse to −20 mV (for Na_V_1.7) or +10 mV (for Na_V_1.8) every 20 s (0.05 Hz). Peptides were diluted in extracellular solution with 0.1% BSA and each peptide concentration was incubated for 5 min. Peak current was normalized to buffer control. The time constant of fast inactivation (τ) was computed by fitting the current decay traces with a single exponential function using the QPatch Assay Software 5.6 (Sophion). *I-V* curves were obtained with a holding potential of −90 mV followed by a series of 500 ms step pulses that ranged from −110 to +55 mV in 5 mV increments (repetition interval 5 s) before and after 5 min incubation or Pme1a (1 μM). Conductance-voltage curves were obtained by calculating the conductance (*G*) at each voltage (*V*) using the equation *G* = *I*/(*V* − *V_rev_*), where *V_rev_* is the reversal potential and were fitted with a Boltzmann equation. 

### 2.8. Data Analysis

Data were plotted and analysed using GraphPad Prism, version 8.2.0. For concentration–response curves, a four-parameter Hill equation with variable Hill coefficient was fitted to the data. Data are presented as the mean ± SEM.

## 3. Results

### 3.1. Isolation of the Novel Spider Venom Peptides μ-TRTX-Pme1a and μ/δ-TRTX-Pme2a from P. metallica

**Figure 1:**
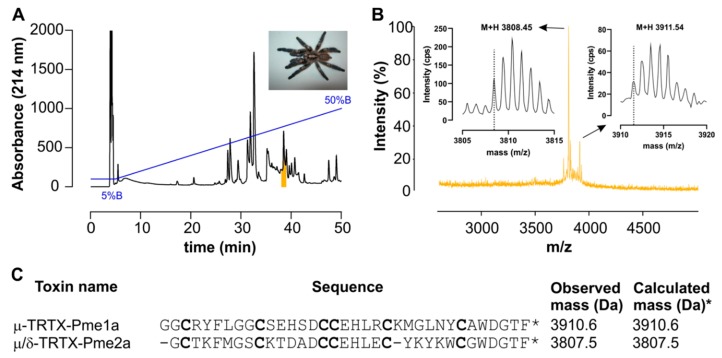
Isolation of novel spider peptides μ-TRTX-Pme1a and μ/δ-TRTX-Pme2a from the venom of *P.* *metallica.* (**A**) Chromatogram resulting from fractionation of the crude venom using RP-HPLC. Blue line indicates gradient of solvent B. Colour indicates the active peak that was further purified using activity guided fraction. (**B**) MALDI-TOF MS spectrum showing the M + H^+^ ions for the dominant masses present in the active peak. (**C**) Sequences of μ-TRTX-Pme1a and δ-TRTX-Pme2a identified by N-terminal sequencing and their observed (uncharged) and calculated (uncharged) monoisotopic masses. The calculated mass was –1 Da due to the amidated C-terminus (as indicated by *).

Crude venom isolated from *P. metallica* (Figure 1A) inhibited deltamethrin-induced membrane potential changes in CHO cells stably expressing hNa_V_1.8, with activity guided fractionation isolating this activity to a single peak eluting at ~35% solvent B (Figure 1A). Matrix-assisted laser desorption/ionization time-of-flight mass spectrometry (MALDI-TOF MS) indicated that this fraction was dominated by two masses (M + H)^+^ of 3911.6 *m*/*z* and 3808.5 *m*/*z* (Figure 1B). N-terminal sequencing revealed two novel 35 and 33 residue peptides that we named μ-TRTX-Pme1a (hereafter Pme1a) and μ/δ-TRTX-Pme2a (hereafter Pme2a) based on the rational nomenclature for peptide toxins (Figure 1C) [19]. The calculated masses and observed masses differed by –1 Da, indicating both Pme1a and Pme2a have an amidated C-terminus. Synthetic Pme1a and Pme2a with C-terminal amidation were used for all further experiments.

### 3.2. Pharmacological Activity of Pme1a

**Figure 2:**
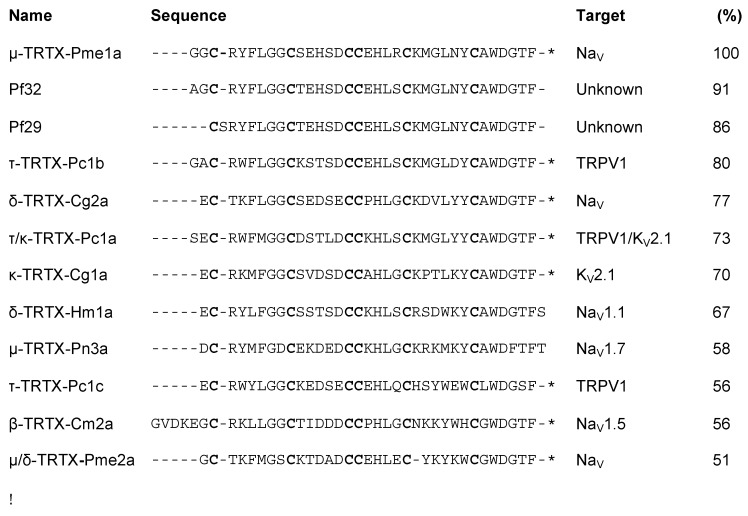
Sequence alignment of Pme1a and Pme2a. Sequence alignment of Pme1a and Pme2a with % sequence identity (to Pme1a) to selected mature spider peptides with a known target from Universal Protein Resource (www.uniprot.org) and two sequences identified from the venom gland of the related species *P. formosa* with unknown activity. Cysteine residues are shown in bold and * indicates amidated C-terminus. Pme1a and Pme2a align to spider peptides belonging to NaSpTx2 family 2.

**Figure 3:**
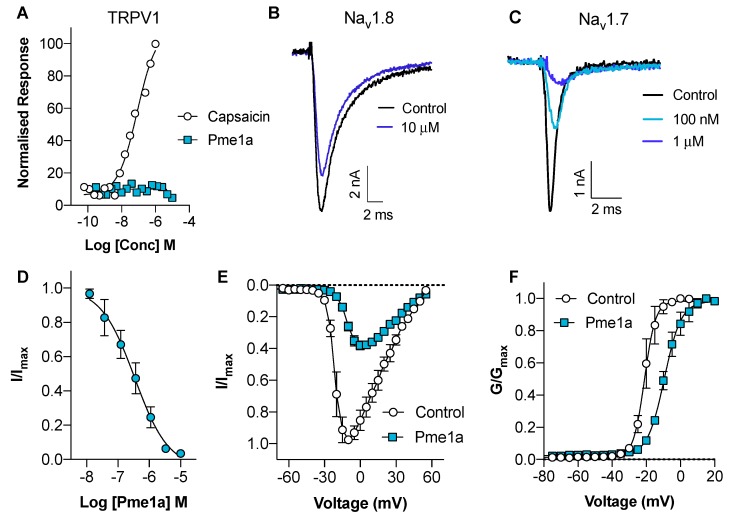
Activity of synthetic Pme1a on TRPV1, Na_V_1.8 and Na_V_1.7. (**A**) Concentration–response curves for capsaicin and Pme1a at rTRPV1 assessed by calcium dye. Data are presented as the mean ± SEM, with *n* = 3–5 wells per data point. (**B**) Representative hNa_V_1.8 current trace before (black) and after addition of Pme1a (blue). Currents were elicited by a 50 ms pulse to +10 mV from a holding potential of −90 mV. (**C**) Representative hNa_V_1.7 current trace before (black) and after addition of Pme1a (blue). Currents were elicited by a 50 ms pulse to −20 mV from a holding potential of −90 mV. (**D**) Concentration–response curve for Pme1a at hNa_V_1.7 (IC_50_ 334 ± 114 nM). (**E**) Current-voltage relationship before (white circles) and after addition of 1 μM Pme1a (blue squares). (**F**) Conductance-voltage curve before (white circles) and after addition of 1 μM Pme1a (blue squares). Pme1a shifted the V_1/2_ of voltage dependence of activation by +11.6 mV. Data are presented as the mean ± SEM, with *n* = 3–6 cells per data point.

Alignment of Pme1a to peptide sequences from the Universal Protein Resource (www.uniprot.org) revealed that the peptide shares high sequence homology (56%–80%) with the vanillotoxins (Figure 2), a family of peptides from *Psalmopoeus cambridgei* that activate TRPV1 with EC_50_s ranging between 0.32 and 12 μM [20]. We therefore tested the activity of Pme1a on TRPV1 heterologously expressed in HEK293 cells. In comparison to the TRPV1 activator capsaicin (EC_50_ 83 nM), Pme1a (up to 30 μM) had no activity on TRPV1, suggesting that amino acid residue(s) crucial for TRPV1 activity are missing from this peptide (Figure 3A). As Pme1a was discovered using a Na_V_ channel screen, we next characterised its activity on Na_V_ channels using automated whole-cell patch-clamp electrophysiology. 

Despite being isolated as an inhibitor of Na_V_1.8 using a fluorescent screen, Pme1a only had modest activity on Nav1.8, inhibiting 21 ± 2 % of the peak current at a concentration of 10 μM (Figure 3B). In contrast, Pme1a was more potent at Na_V_1.7, inhibiting peak current with an IC_50_ of 334 ± 114 nM (Figure 3C,D). Activity at other Na_V_ channel subtypes is not unexpected, given the high-sequence homology between Na_V_ subtypes [12]. To examine the mechanism of channel block, we next assessed the effect of Pme1a on the voltage-current relationship at Na_V_1.7 (Figure 3E). Pme1a (1 μM) shifted the voltage dependence of activation at Na_V_1.7 to more depolarised membrane potentials (V_1/2_ activation: Δ = +11.6 mV), confirming it binds to the voltage-sensing domain(s) to modify channel gating (Figure 3F). 

### 3.3. Pharmacological Activity of Pme2a

**Figure 4:**
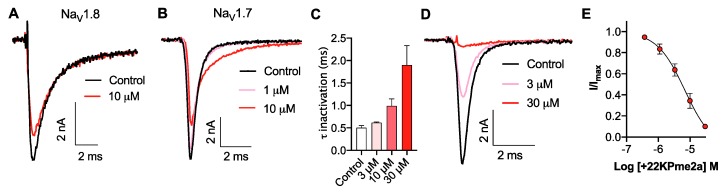
Activity of synthetic Pme2a and [+22K]Pme2a on Na_V_1.8 and Na_V_1.7. (**A**) Representative hNa_V_1.8 current trace before (black) and after addition of Pme2a (red). Currents were elicited by a 50 ms pulse to +10 mV from a holding potential of −90 mV. (**B**) Representative hNa_V_1.7 current trace before (black) and after addition of Pme2a (red). Currents were elicited by a 50 ms pulse to −20 mV from a holding potential of −90 mV. (**C**) Pme2a concentration-dependently increases the time constant of fast inactivation (τ) at hNa_V_1.7. (**D**) Representative hNa_V_1.7 current trace before (black) and after addition of [+22K]Pme2a (red). Currents were elicited by a 50 ms pulse to −20 mV from a holding potential of −90 mV. (**E**) Concentration–response curve for [+22K]Pme2a at hNa_V_1.7 (IC_50_ 5.6 ± 1.1 μM). Data are presented as the mean ± SEM, with *n* = 4 cells per data point.

Alignment of Pme2a to peptide sequences from the Universal Protein Resource (www.uniprot.org) revealed that the peptide shares sequence homology (<65%) to Pme1a and other peptides with activity at Na_V_ channels (Figure 2). Similar to Pme1a, Pme2a had no activity at TRPV1 (data not shown), and only modest activity at Nav1.8, with the highest concentration tested (10 μM) inhibiting 22 ± 4 % of peak current (Figure 4A). Pme2a also had modest activity at Na_V_1.7, concentration-dependently delaying-fast inactivation, albeit not potently, with an estimated EC_50_ > 10 μM (Figure 4B,C). Due to low potency at Na_V_1.7, no further pharmacological characterisation was performed. 

Sequence alignment revealed that loop 4 of Pme2a is one amino acid residue shorter compared to others in the family, and peptides with activity on TTX-sensitive Na_V_ channels generally contain a positively charged amino acid at the equivalent position, most often a lysine (Figure 2). We therefore synthesised a [+22K]Pme2a mutant and assessed how the addition of a lysine affected activity at Na_V_1.7. [+22K]Pme2a concentration-dependently inhibited Na_V_1.7 peak current, without the delay in fast activation seen with the native peptide, with a more potent IC_50_ of 5.6 ± 1.1 μM (Figure 4D,E).

## 4. Discussion

Here, we describe the isolation and Na_V_ activity of two novel spider venom-derived peptides, which are the first peptides to be isolated and functionally characterised from the venom of the species *P. metallica,* as well as the entire *Poecilotheria* genus [21]. A transcriptome and proteome from the venom gland of the related species *P. formosa* identified two related, but not identical peptide sequences (Pf29 and Pf32) [22], indicating that the entire *Poecilotheria* genus is likely to be a rich source of novel Na_V_ modulators (Figure 2). Indeed, crude venom from several species of the *Poecilotheria* genus, including *P. metallica,* has previously been shown to modulate Na_V_1.7 [23], consistent with activity of Pme1a and Pme2a at both Na_V_1.7 and Na_V_1.8 described here. The two peptides isolated here were only moderately potent at Na_V_1.7 and Na_V_1.8, indicating that there may still be additional peptides in the crude venom with more potent activity.

Specifically, both Pme1a and Pme2a align with NaSpTx family 2, which is a large family of spider venom peptides that have promiscuous activity on Na_V_, K_V_ and Ca_V_ channels [24]. Interestingly, this family also contains members that have high selectivity for Na_V_ channels of therapeutic interest, including the Na_V_1.7 inhibitor μ-TRTX-Pn3a and the Na_V_1.1 activator δ-TRTX-Hm1a. However, little is known about the structure–activity relationships that define the pharmacology of this family [25,26]. Here, we describe the importance of a lysine (K) at position “22” (using Pme2a numbering) for Na_V_ channel activity. The presence of the positively charged amino acid residue lysine is often important for facilitating membrane interactions in venom-based peptides [18,27]. Addition of K22 altered the pharmacology of Pme2a, converting the native peptide from being a Na_V_1.7 channel activator to a more potent Na_V_1.7 channel inhibitor. This is a significant finding, indicating that the amino acid residue(s) present at or around this site can dictate whether family 2 peptides function as Na_V_ activators or inhibitors. It is also possible that extending loop 4 of Pme2a by one amino acid residue contributed to the change in activity by slightly altering the overall structure of the peptide. Future studies elucidating the full Na_V_ selectivity of Pme1a and Pme2a will further define which amino acid residues are important for Na_V_ channel potency and subtype selectivity.

In conclusion, we have identified two novel spider venom-derived peptides that modulate Na_V_ channels. Results from this study provide structure–activity relationship information that may guide the future rational design of spider venom-derived peptides with improved activity and selectivity at Na_V_ channels.
